# Association between coronary artery calcium score and myocardial ischemia assessed by stress perfusion cardiac magnetic resonance imaging

**DOI:** 10.1371/journal.pone.0361659

**Published:** 2026-10-30

**Authors:** Yodying Kaolawanich, Methat Meechuen, Issarayus Laohabut, Thanawat Ruengchaisiwawaith, Thananya Boonyasirinant

**Affiliations:** Division of Cardiology, Department of Medicine, Faculty of Medicine Siriraj Hospital, Mahidol University, Bangkok, Thailand; CUNY School of Medicine: The City College of New York CUNY School of Medicine, UNITED STATES OF AMERICA

## Abstract

**Background:**

Coronary artery calcium (CAC) scoring effectively stratifies cardiovascular risk, and stress perfusion cardiac magnetic resonance (CMR) accurately detects myocardial ischemia. We aimed to investigate the prevalence and factors associated with myocardial ischemia on CMR in patients with CAC, and to assess the clinical impact of myocardial ischemia following CMR.

**Methods:**

This study included 215 patients aged ≥18 years who underwent CAC scoring for risk stratification, followed by stress CMR between 2013 and 2026. The prevalence and predictors of myocardial ischemia were investigated. Receiver operating characteristic (ROC) analysis was performed to assess the association between CAC scores and myocardial ischemia. Additionally, subsequent clinical management following CMR was determined.

**Results:**

The mean age was 69.3 ± 9.1 years (60.9% male). The average 10-year PREVENT risk score was 18.6 ± 8.7%. Overall, myocardial ischemia was present in 26.0% of patients (56/215): 17.1% in the CAC 300–999 Agatston units (AU) group (14/82) and 41.6% in the CAC ≥ 1,000 AU group (42/101). CAC score was the only independent predictor of myocardial ischemia (adjusted OR 1.07, 95%CI 1.03 − 1.10; p < 0.001 per 100 AU). Across clinical risk score categories and diabetes status, CAC score was consistently associated with myocardial ischemia, with no significant interactions observed (all p for interaction >0.05).The number of ischemic segments increased with increasing CAC scores (p < 0.001). ROC analysis demonstrated an optimal cutoff threshold of CAC ≥ 1,093 AU for detecting myocardial ischemia (AUC 0.75, p < 0.001). The clinical impact of stress CMR included medication changes in 37.2% of patients, while 18.1% underwent invasive procedures.

**Conclusions:**

In patients with CAC scores undergoing stress CMR, the prevalence of myocardial ischemia was approximately one-fourth and was associated with higher CAC scores. Stress CMR may influence clinical management in this population. Larger or prospective studies validating these findings and evaluating prognosis are warranted.

## Introduction

Coronary artery calcium (CAC) scoring is an established, noninvasive marker of coronary atherosclerotic burden and a strong predictor of cardiovascular risk [1–3]. Patients with high CAC scores, particularly ≥400 or ≥1,000 Agatston unit (AU), have a markedly increased risk of major adverse cardiovascular events [4]. However, CAC reflects total plaque burden rather than hemodynamic significance, and it does not directly identify myocardial ischemia [5–9].

Despite the increasing use of CAC screening, the clinical utility of downstream stress testing in asymptomatic or minimally symptomatic individuals with high CAC scores is of interest. Several studies have investigated the assessment of myocardial ischemia using single-photon emission computed tomography (SPECT) or positron emission tomography (PET) perfusion imaging in patients with CAC [10–12]. A meta-analysis by Bavishi et al., including 20 SPECT studies indicates the presence of a quantitative relationship between the magnitude of CAC and the likelihood of inducible myocardial ischemia [13].

In asymptomatic patients with elevated CAC, while established modalities like SPECT and PET offer proven clinical utility, stress perfusion cardiac magnetic resonance (CMR) provides a radiation-free alternative with high spatial resolution, capable of detecting subtle myocardial ischemia [14]. Additionally, stress perfusion CMR uniquely integrates stress testing with late gadolinium enhancement (LGE) in a single examination, enabling simultaneous assessment of inducible ischemia and myocardial scar to optimize risk stratification [15,16].

There was some evidence regarding stress perfusion CMR in patients with CAC. Rijlaarsdam-Hermsen, et al. demonstrated that stress-only perfusion CMR serves as a highly accurate gatekeeper for stable chest pain patients with high CAC scores, offering high specificity and negative predictive value to safely defer unnecessary invasive testing [17]. However, evidence regarding stress perfusion CMR in patients who are asymptomatic with risk factors and a positive CAC score is limited.

We hypothesized that higher CAC scores are associated with myocardial ischemia on stress perfusion CMR, and that CMR findings may be associated with subsequent changes in clinical management, such as medication adjustments or invasive procedures. Accordingly, the aim of this study was to evaluate the prevalence and predictors of myocardial ischemia on stress perfusion CMR in patients with CAC, and to assess the clinical impact of CMR on subsequent management.

## Methods

### Study population

This retrospective cohort study included consecutive patients aged 18 years or older who underwent CAC scoring for screening and cardiovascular risk stratification, had detectable CAC, and subsequently underwent stress perfusion CMR between November 2013 and May 2026 at the Division of Cardiology, Department of Medicine, Faculty of Medicine Siriraj Hospital, Mahidol University, Bangkok, Thailand. Exclusion criteria included a duration between computed tomography (CT) and CMR of >180 days, CMR findings revealing specific non-ischemic cardiomyopathies (e.g., hypertrophic or infiltrative cardiomyopathy), congenital heart disease, and incomplete CT or CMR data.

Clinical characteristics, medications, and laboratory results were collected on the day of CMR. Two clinical 10-year cardiovascular (CV) risk scores were evaluated: the Predicting Risk of Cardiovascular Disease EVENTs (PREVENT) equations [18]–categorized into low/borderline risk (<5%), intermediate risk (5% to <10%), and high risk (≥10%)–and the Thai CV risk score, which was developed and validated to estimate CV disease risk specifically within the Thai population [19,20], categorized into low risk (<10%), intermediate risk (10% to <20%), and high/very high risk (≥20%).

The study protocol conformed to the ethical guidelines of the Declaration of Helsinki. This study was approved by the Siriraj Institutional Review Board (SIRB), Faculty of Medicine Siriraj Hospital, Mahidol University (COA no. Si 560/2026). The requirement for informed consent was waived by the board because of the retrospective study design and the removal of all personal identifying information. Data were accessed for research purposes between July 1 and July 31, 2026.

### CT protocol and image analysis

Non-contrast CT was performed using a 256-slice scanner (SOMATOM Definition Flash; Siemens Healthcare, Erlangen, Germany) according to established guidelines and institutional protocol at the time of the scan [21]. A prospective electrocardiography (ECG)-gated sequential CT scan was performed to determine CAC scoring using the following parameters: rotation time of 280 ms, slice collimation of 0.6 mm, slice width of 3.0 mm, tube voltage of 120 kV, and tube current of 50 mAs [22]. Analysis of CAC images was performed following the standard protocol on a separate workstation (Syngovia; Siemens Healthineers, Erlangen, Germany). CAC scans were interpreted according to the Agatston method [23].

### CMR protocol and image analysis

The CMR studies were performed with a 1.5 or 3.0 Tesla (T) Philips scanner (Philips Medical Systems, Best, The Netherlands). Details of the CMR protocol and parameters, including functional assessment, adenosine stress perfusion, and LGE, are provided in the Supporting information (S1 File).

All CMR image interpretations were performed by CMR-trained cardiologists according to standard SCMR criteria. Quantitative measurements of left ventricular (LV) volumes, left ventricular ejection fraction (LVEF), and LV mass were obtained from a stack of short-axis steady-state free precession cine images [24]. Perfusion and LGE images were evaluated using visual assessment. Perfusion images were examined, with visualization of each of the 16 segments (excluding segment-17 at the apex). Inducible ischemia was characterized as a subendocardial perfusion defect meeting specific criteria: persistence beyond peak myocardial enhancement and for several RR intervals, width exceeding two pixels, following one or more coronary arteries, and absence of LGE in the corresponding segment [24]. LGE was considered present only when confirmed in both the short-axis view and at least one other orthogonal plane [24]. The total number of ischemic and LGE segments was determined utilizing the American Heart Association 17-segment model [25].

### Subsequent clinical management following CMR

To assess the clinical impact of stress perfusion CMR on patient management, two cardiologists independently reviewed electronic medical records up to the first subsequent outpatient visit (last follow-up date: June 10, 2026). Changes in management were defined as documented treatment modifications made at this visit, specifically: (1) medical therapy adjustments (initiation, titration, or discontinuation of medications), and (2) referrals for invasive procedures, including coronary angiography and revascularization.

### Statistical analysis

All statistical analyses were performed using SPSS software, version 20.0 (IBM Corp., Armonk, NY, USA). Continuous variables are presented as mean ± standard deviation or median (interquartile range [IQR]), as appropriate, and categorical variables as frequencies and percentages. Comparisons between groups were performed using the Student’s t-test or Mann–Whitney U test for continuous variables and the chi-square or Fisher’s exact test for categorical variables, as appropriate. Univariable and multivariable logistic regression analyses were used to identify factors associated with myocardial ischemia, with results reported as unadjusted and adjusted odds ratios (ORs) and 95% confidence intervals (CIs). Two logistic regression models were utilized: the first model evaluated CAC as a continuous variable, and the second model evaluated CAC using established cutoff values. Sensitivity analyses evaluating the association between CAC score and myocardial ischemia stratified by clinical risk score categories and diabetes mellitus status were also performed. Receiver operating characteristic (ROC) curve analysis was performed to evaluate the predictive performance of the CAC score for myocardial ischemia. The area under the curve (AUC) was calculated, and sensitivity and specificity were derived across cutoff values, with the optimal cutoff determined using the Youden index. The correlation between the CAC score and the number of ischemic segments was also evaluated using Spearman’s rank correlation test.

## Result

### Patient characteristics

Fig 1 demonstrates the study flowchart. A total of 280 patients with CAC who subsequently underwent stress perfusion CMR were screened for eligibility. Of these, 57 were excluded due to a duration between CT and CMR of >180 days, and 4 were excluded due to incomplete CT or CMR data. Following CMR, an additional 4 patients were excluded due to specific non-ischemic cardiomyopathy on CMR. Finally, 215 patients were included for the analysis.

**Fig 1:**
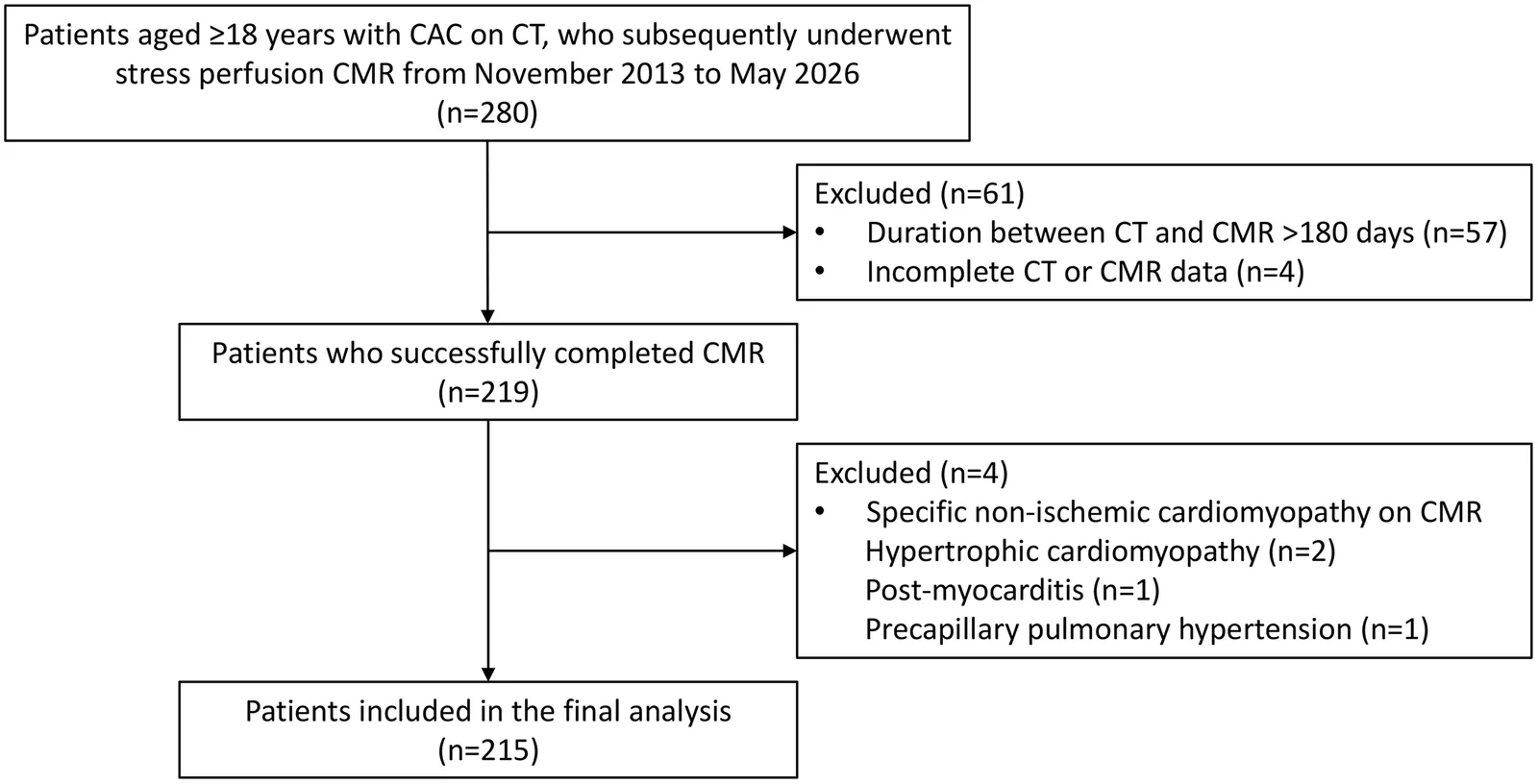
A flow diagram describing the patient enrollment protocol for this study. **Abbreviations:** CAC, coronary artery calcium; CT, computed tomography; CMR, cardiac magnetic resonance.

The median duration between CT and CMR was 48 (12–76) days. Table 1 shows the clinical and imaging characteristics of the study population at the time of CMR, stratified by the presence or absence of myocardial ischemia. Overall, the mean age was 69.3 ± 9.1 years, and 131 (60.9%) patients were male. Hypertension and diabetes mellitus were present in 161 (74.9%) and 72 (33.5%) patients, respectively. The mean PREVENT and Thai CV risk scores were 18.6% ± 8.7% and 23.6% ± 7.6%, respectively. Regarding CAC score categories, 5.6% of patients had CAC 1–99 AU, 9.3% had CAC 100–299 AU, 38.1% had CAC 300–999 AU, and 47.0% had CAC ≥ 1,000 AU.

**Table 1:** Clinical and imaging characteristics of the study population at the time of CMR, stratified by the presence or absence of myocardial ischemia.

	Total	Ischemia presence	Ischemia absence	P-value
	(n = 215)	(n = 56)	(n = 159)	
Age, years	69.3 ± 9.1	69.6 ± 9.4	69.2 ± 9.0	0.79
Male	131 (60.9)	42 (75.0)	89 (56.0)	***0.01***
BMI, kg/m^2^	24.8 ± 4.3	25.1 ± 4.2	24.7 ± 4.3	0.48
Systolic BP, mmHg	137.8 ± 19.3	136.5 ± 14.9	138.3 ± 20.6	0.48
Diastolic BP, mmHg	73.6 ± 11.1	72.7 ± 11.2	73.9 ± 11.1	0.47
Heart rate, bpm	80.2 ± 14.7	79.8 ± 15.1	80.3 ± 14.6	0.82
CAD risk factors				
Hypertension	161 (74.9)	38 (67.9)	123 (77.4)	0.15
Diabetes mellitus	72 (33.5)	17 (30.4)	55 (34.6)	0.56
Hyperlipidemia	174 (80.9)	46 (82.1)	128 (80.5)	0.78
Family history of CAD	5 (2.3)	1 (1.8)	4 (2.5)	0.75
Current smoker	8 (3.7)	0 (0.0)	8 (5.0)	0.08
Former smoker	5 (2.3)	0 (0.0)	5 (3.1)	0.33
Medications				
Antiplatelet	94 (43.7)	21 (37.5)	73 (45.9)	0.34
ACE inhibitor or ARB	72 (33.5)	19 (33.9)	53 (33.3)	0.93
Beta blocker	73 (34.0)	16 (28.6)	57 (35.8)	0.32
Calcium channel blocker	55 (25.6)	13 (23.2)	42 (26.4)	0.63
Statin	149 (69.3)	37 (66.1)	112 (70.4)	0.54
Laboratory results				
Total cholesterol, mg/dL	166.5 ± 40.7	161.4 ± 39.3	168.2 ± 41.1	0.32
Triglycerides, mg/dL	120.5 ± 73.1	129.0 ± 104.7	117.5 ± 58.3	0.34
HDL cholesterol, mg/dL	56.1 ± 16.9	54.5 ± 22.3	56.6 ± 14.7	0.45
LDL cholesterol, mg/dL	87.7 ± 37.8	85.2 ± 36.7	88.6 ± 38.3	0.60
eGFR, mL/min/1.73 m²	78.3 ± 18.7	76.1 ± 18.7	79.1 ± 18.7	0.33
PREVENT risk score, mean ± SD, % ^a^	18.6 ± 8.7	19.4 ± 9.2	18.3 ± 8.6	0.44
PREVENT risk score category, n (%) ^a^				0.90
Low/borderline risk	8 (4.4)	2 (4.3)	6 (4.5)	
Intermediate risk	27 (15.0)	6 (13.0)	21 (15.7)	
High risk	145 (80.6)	38 (82.6)	107 (79.9)	
Thai CV risk score, mean ± SD, % ^b^	23.6 ± 7.6	23.6 ± 7.7	23.7 ± 7.6	0.95
Thai CV risk score category, n (%) ^b^				0.93
Low risk	12 (6.5)	3 (6.2)	9 (6.5)	
Intermediate risk	42 (22.6)	10 (20.8)	32 (23.2)	
High/very high risk	132 (71.0)	35 (72.9)	97 (70.3)	
CT				
Total CAC score, Agatston units	919 (511–1,667)	1,419.5 (1,032.5–2,111.5)	734 (393–1,358)	***<0.001***
CAC score categories, n (%)				***<0.001***
1–99	12 (5.6)	0 (0.0)	12 (7.5)	
100-299	20 (9.3)	0 (0.0)	20 (12.6)	
300–999	82 (38.1)	14 (25.0)	68 (42.8)	
≥ 1,000	101 (47.0)	42 (75.0)	59 (37.1)	
CMR				
LVEDV index, mL/m²	67.6 ± 12.6	67.3 ± 12.8	67.7 ± 12.6	0.86
LVESV index, mL/m²	19.5 ± 7.7	19.9 ± 8.7	19.4 ± 7.3	0.68
LV mass index, g/m^2^	40.1 ± 9.9	41.2 ± 9.5	39.7 ± 10.0	0.34
LVEF, %	71.6 ± 7.8	70.9 ± 9.3	71.8 ± 7.2	0.47
Number of ischemic segments ^c^	5 (3–9)	5 (3–9)	0 (0–0)	***<0.001***
Presence of LGE	21 (9.8)	18 (32.1)	3 (1.9)	***<0.001***
Number of LGE segments ^d^	3 (1–4)	3 (1–4)	2 (1–3.5)	0.60

A p-value<0.05 indicates statistical significance.

^a^PREVENT risk score available for n = 180.

^b^Thai CV risk score available for n = 186.

^c^In patients with myocardial ischemia.

^d^In patients with LGE. Abbreviations: ACE, angiotensin-converting enzyme; ARB, angiotensin receptor blocker; BMI, body mass index; BP, blood pressure; CAC, coronary artery calcium; CAD, coronary artery disease; CMR, cardiac magnetic resonance; CT, computed tomography; CV, cardiovascular; eGFR, estimated glomerular filtration rate; HDL, high-density lipoprotein; LDL, low-density lipoprotein; LGE, late gadolinium enhancement; LV, left ventricular; LVEDV, left ventricular end-diastolic volume; LVEF, left ventricular ejection fraction; LVESV, left ventricular end-systolic volume; PREVENT, Predicting Risk of Cardiovascular Disease EVENTs.

### Prevalence of myocardial ischemia

Fifty-six (26.0%) patients had myocardial ischemia on CMR imaging, with a median number of ischemic segments of 5 (IQR 3–9) segments (Table 1). Compared with patients without myocardial ischemia, those with myocardial ischemia showed no significant differences in clinical characteristics, except that patients with ischemia were more likely to be male (75.0% versus 56.0%, p = 0.01). There were also no significant differences in medication use, laboratory results, or PREVENT and Thai CV risk scores (all p > 0.05) (Table 1). Regarding CMR parameters, patients with myocardial ischemia had a significantly higher rate of LGE (32.1% versus 1.9%, p < 0.001). LV volume indices, LV mass index, and LVEF were not significantly different between the groups (all p > 0.05).

Fig 2A demonstrates the prevalence of myocardial ischemia across different CAC score categories. Among patients with a CAC score <300 AU (n = 32), no myocardial ischemia was identified on stress perfusion CMR (0.0%, 95% CI 0.0%–10.9%). Patients with a CAC score of 300–999 AU (n = 82) demonstrated an ischemia rate of 17.1% (95% CI 9.7%–27.0%), whereas those with a CAC score ≥1,000 AU (n = 101) had a rate of 41.6% (95% CI 31.9%–51.8%). Fig 2B demonstrates the number of ischemic segments across CAC score categories. There was a progressive increase in the percentage of patients with >2 ischemic segments as the CAC score increased. Among patients with a CAC score of 300–999 AU, 3.7% had 1–2 ischemic segments and 13.4% had > 2 ischemic segments (p < 0.001). Among those with a CAC score ≥1,000 AU, 5.0% had 1–2 ischemic segments and 36.6% had > 2 ischemic segments (p < 0.001).

**Fig 2:**
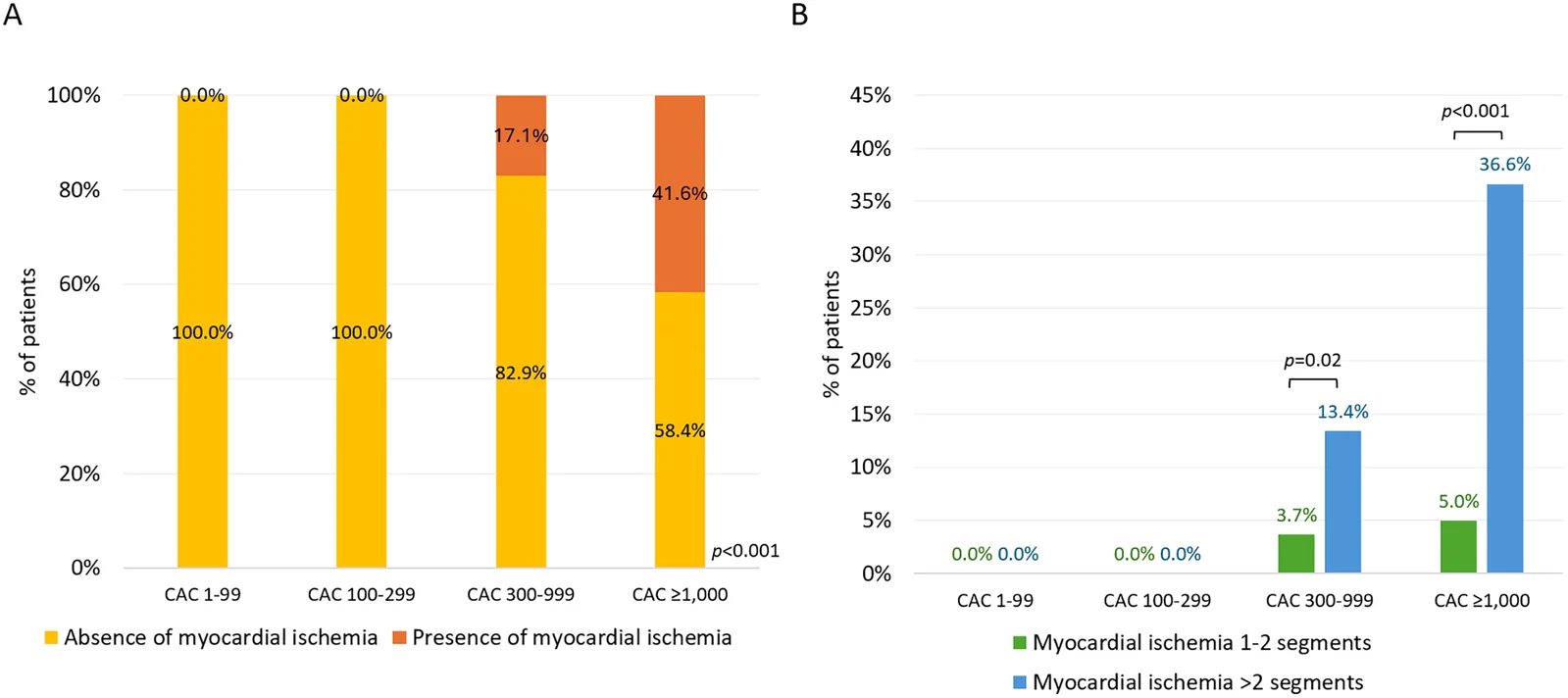
Distribution of patients with myocardial ischemia across 4 different CAC score categories, showing (A) the proportions of patients without and with myocardial ischemia, and (B) patients with myocardial ischemia in 1-2 segments versus patients with myocardial ischemia in >2 segments. **Abbreviations:** CAC, coronary artery calcium.

### Factors associated with myocardial ischemia

Table 2 shows the univariable and multivariable binary logistic regression analyses of factors associated with myocardial ischemia. In the univariable analysis, male sex (OR 2.36, 95% CI 1.19–4.66, p = 0.01), total CAC score per 100 AU (OR 1.07, 95% CI 1.04–1.11, p < 0.001), and a total CAC score ≥1,000 AU (OR 4.95, 95% CI 2.49–9.81, p < 0.001) were significantly associated with myocardial ischemia. In the multivariable analysis, only the CAC score was independently associated with myocardial ischemia. This was observed in Model 1 using the continuous CAC score (adjusted OR [aOR] 1.07, 95% CI 1.03–1.10, p < 0.001) and in Model 2 using the categorical CAC score (aOR 4.53, 95% CI 2.26–9.05, p < 0.001).

**Table 2:** Univariable and multivariable binary logistic regression analysis of factors associated with myocardial ischemia.

	Univariable analysis			Multivariable analysis					
				Model 1 ^a^			Model 2 ^b^		
	OR	95% CI	P-value	aOR	95% CI	P-value	aOR	95% CI	P-value
Age	1.005	0.97–1.04	0.79						
Male	2.36	1.19–4.66	***0.01***	1.92	0.95–3.91	0.07	1.89	0.92–3.87	0.08
BMI	1.02	0.95–1.09	0.48						
Systolic BP	0.99	0.97–1.01	0.54						
Diastolic BP	0.99	0.96–1.01	0.47						
Heart rate	0.99	0.97–1.02	0.82						
Hypertension	0.61	0.31–1.21	0.16						
Diabetes mellitus	0.82	0.42–1.58	0.56						
Hyperlipidemia	1.11	0.50–2.45	0.78						
Family history of CAD	0.75	0.08–6.44	0.70						
Antiplatelet	0.71	0.38–1.32	0.27						
ACE inhibitor or ARB	1.02	0.53–1.95	0.93						
Beta blocker	0.72	0.36–1.39	0.32						
Calcium channel blocker	0.84	0.41–1.71	0.63						
Statin	0.81	0.42–1.56	0.54						
Total cholesterol	1.007	0.99–1.01	0.24						
Triglycerides	1.003	0.99–1.008	0.23						
HDL cholesterol	0.99	0.97–1.02	0.72						
LDL cholesterol	1.01	0.99–1.02	0.10						
eGFR	0.99	0.97–1.008	0.33						
PREVENT risk scores (%)	1.01	0.97–1.05	0.44						
Thai CV risk scores (%)	1.00	0.95–1.04	0.95						
Total CAC score (per 100 Agatston units)	1.07	1.04–1.11	***<0.001***	1.07	1.03–1.10	***<0.001***			
Total CAC score ≥1,000 Agatston units	4.95	2.49–9.81	***<0.001***				4.53	2.26–9.05	***<0.001***

A p-value<0.05 indicates statistical significance.

^a^Model 1 included total CAC sore as continuous variables.

^b^Model 2 included total CAC sore as categorical variables.

Abbreviations: aOR, adjusted odd ratio; ACE, angiotensin-converting enzyme; ARB, angiotensin receptor blocker; BMI, body mass index; BP, blood pressure; bpm, beats per minute; CAC, coronary artery calcium; CAD, coronary artery disease; CI, confidence interval; CV, cardiovascular; eGFR, estimated glomerular filtration rate; HDL, high-density lipoprotein; LDL, low-density lipoprotein; OR, odd ratio; PREVENT, Predicting Risk of Cardiovascular Disease EVENTs.

Table 3 shows the ORs of the total CAC score (per 100 AU) associated with myocardial ischemia across different PREVENT and Thai CV risk score categories (low, intermediate, and high risk). For both risk scores, higher ORs were observed with increasing CAC scores, with no heterogeneity across the categories (p for interaction = 0.89 for PREVENT and 0.27 for the Thai CV risk score).

**Table 3:** Odds ratio of total CAC scores (per 100 Agatston units) associated with myocardial ischemia across clinical risk strata for the PREVENT and Thai CV risk score assessment tools.

Clinical risk assessment models	Odds ratio	95% CI	P-value	P-value for interaction
**PREVENT risk score**				0.89
Low/borderline risk	1.003	0.92-1.10	0.94	
Intermediate risk	1.25	1.03-1.51	***0.04***	
High risk	1.07	1.03-1.11	***0.001***	
**Thai CV risk score**				0.27
Low risk	1.009	0.93-1.10	0.83	
Intermediate risk	1.12	0.99-1.26	0.06	
High/very high risk	1.07	1.03-1.12	***0.001***	

A p-value<0.05 indicates statistical significance.

Abbreviations: CI, confidence interval; CAC, coronary artery calcium; CV, cardiovascular; PREVENT, Predicting Risk of Cardiovascular Disease EVENTs.

In a sensitivity analysis stratified by diabetes status, patients with diabetes had significantly higher CAC scores than those without diabetes (1,375.5 [IQR 649.5–2,144.4] versus 800 [IQR 452–1,337] AU, p < 0.001), but there were no significant differences in the prevalence of myocardial ischemia (17/72 [23.6%] versus 39/143 [27.3%], p = 0.56) or the number of ischemic segments (5 [IQR 4–8] versus 5 [IQR 3–9] segments, respectively, p = 0.95) (S1 Table). Overall, CAC score remained significantly associated with myocardial ischemia, with no significant interaction by diabetes status (p for interaction = 0.94).

### ROC and correlation analysis

Fig 3 shows the ROC analysis of the CAC score to predict myocardial ischemia, yielding an AUC of 0.75 (95% CI 0.68–0.81, p < 0.001). An optimal CAC score cutoff of ≥1,093 AU predicted myocardial ischemia with a sensitivity of 75.0%, a specificity of 69.2%, and a Youden’s index of 0.44.

**Fig 3:**
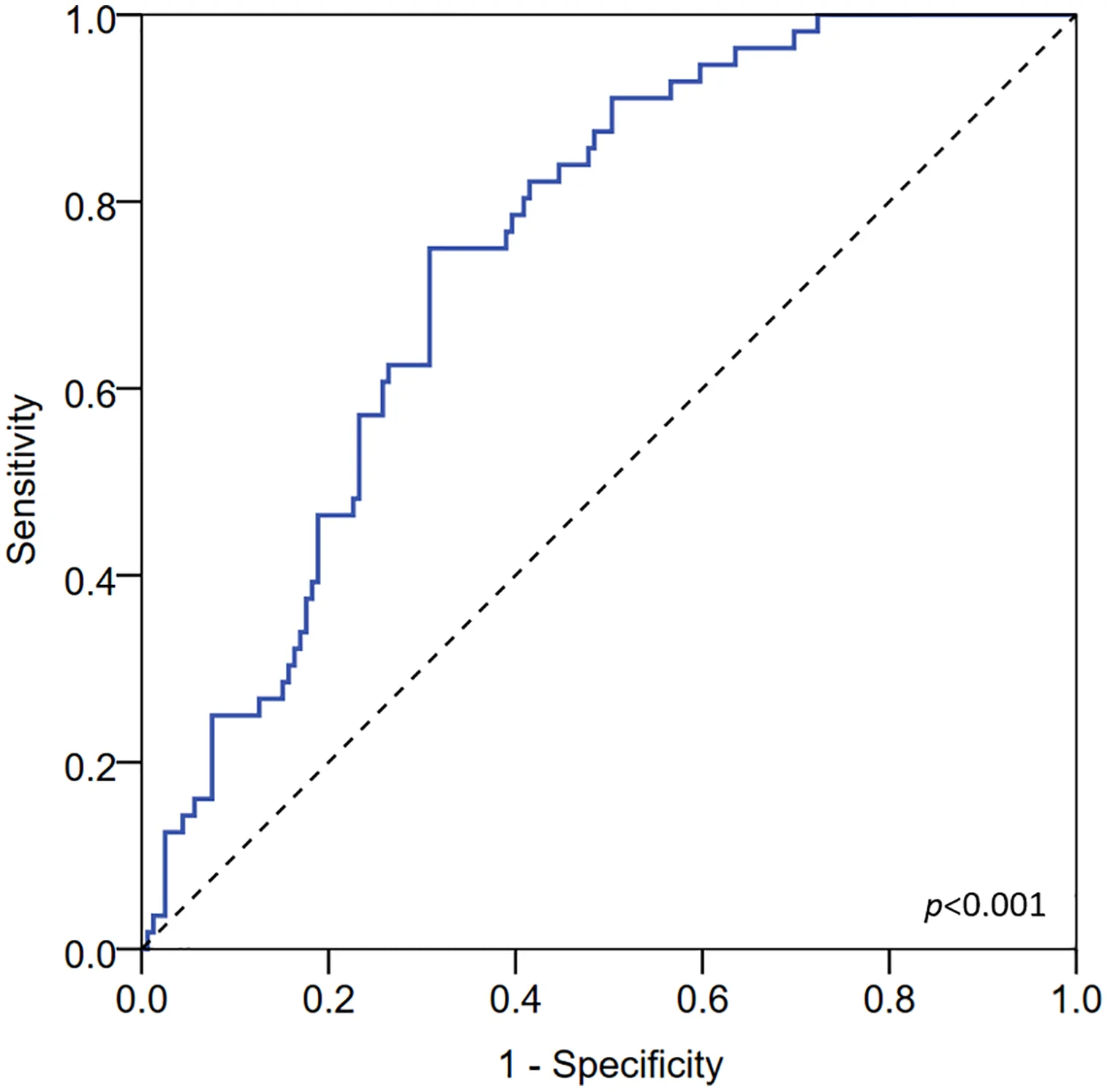
Receiver operating characteristic curve analysis of the CAC score for predicting the presence of myocardial ischemia. A CAC score of ≥1,093 Agatston units was identified as the optimal cutoff to predict the presence of myocardial ischemia (area under the curve 0.75, p < 0.001). **Abbreviations:** CAC, coronary artery calcium.

In a sensitivity analysis stratified by diabetes status, the AUC of the CAC score for predicting myocardial ischemia was not significantly different between patients with and without diabetes (AUC 0.76, 95% CI 0.64–0.89 versus AUC 0.77, 95% CI 0.70–0.86, respectively, p = 0.79). However, patients with diabetes demonstrated a higher optimal cutoff threshold than those without diabetes (diabetes: ≥ 1,590 AU [sensitivity 76.5%, specificity 69.1%, Youden index 0.46] vs. non-diabetes: ≥ 1,093 AU [sensitivity 69.2%, specificity 79.8%, Youden index 0.49]).

Fig 4 shows the correlation between the CAC score and the number of ischemic segments. There was a statistically significant, weak-to-moderate positive correlation between the CAC score and the number of ischemic myocardial segments (r = 0.37, p < 0.001).

**Fig 4:**
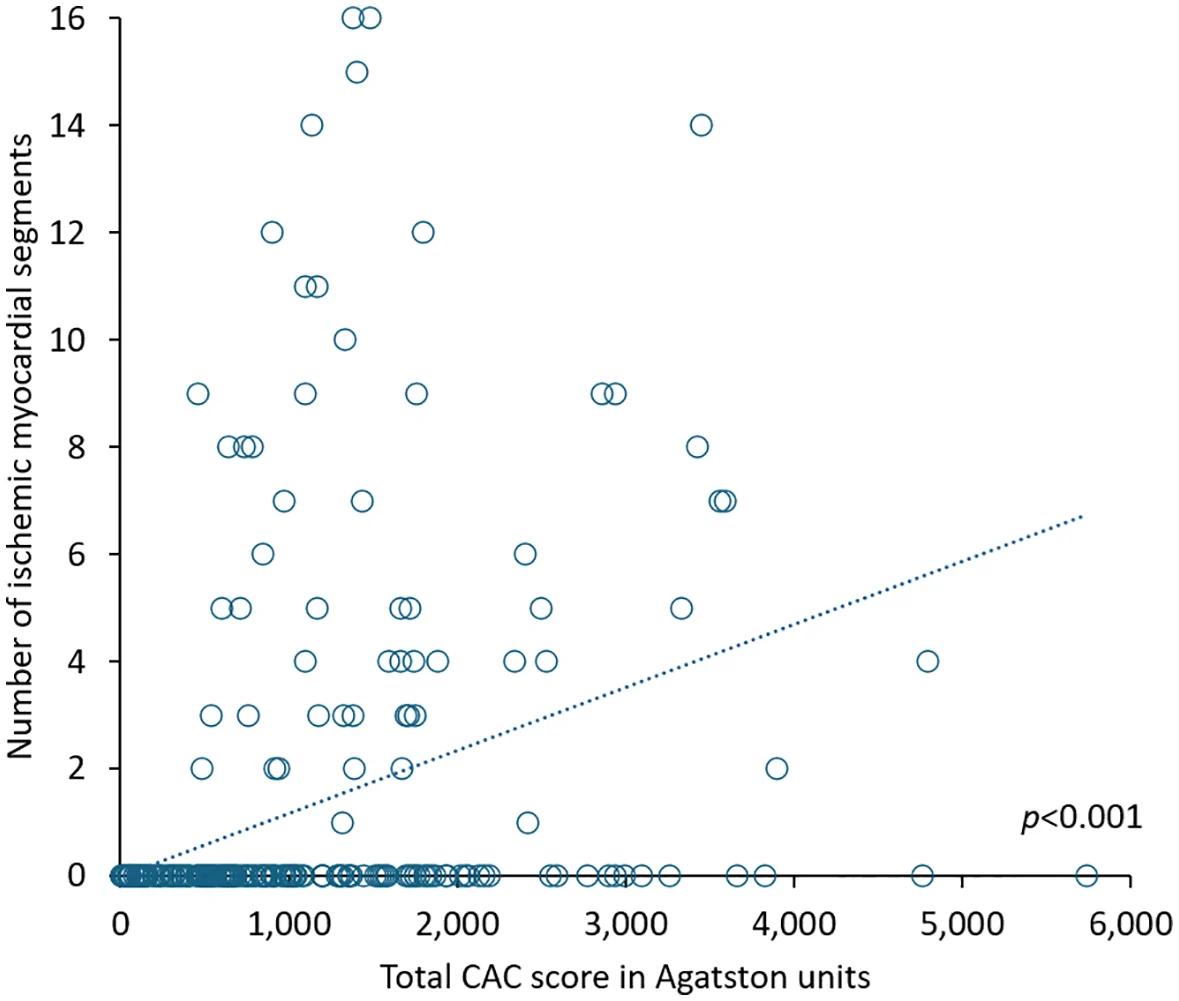
Correlation between the CAC score and the number of ischemic myocardial segments. There was a statistically significant weak-to-moderate positive correlation between the CAC score and the number of ischemic myocardial segments (r = 0.37, p < 0.001), which indicates a higher calcium burden to be significantly associated with a greater extent of myocardial ischemia. **Abbreviations:** CAC, coronary artery calcium.

### Subsequent clinical management following CMR

Table 4 shows the clinical impact of stress perfusion CMR. After CMR, 80 (37.2%) of patients had a medication change, including 61 (28.4%) who had addition of a new medication or an increase in the dose of a current medication, most commonly antiplatelets (n = 42, 19.5%) and statins (n = 30, 14.0%). Additionally, 39 (18.1%) patients underwent invasive coronary angiography, after which 27 underwent percutaneous coronary intervention and 10 underwent coronary artery bypass graft surgery. Notably, all 39 of these patients belonged to the myocardial ischemia group.

**Table 4:** The clinical impact of stress perfusion CMR.

Types of clinical impact	n (%)
Patients whose medication was changed	80 (37.2)
Addition of a new medication or an increase in the dose of a current medication	61 (28.4)
Antiplatelet	42 (19.5)
ACE inhibitor or ARB	10 (4.7)
Beta blocker	10 (4.7)
Calcium channel blocker	3 (1.4)
Statin	30 (14.0)
Discontinuation of a medication	9 (4.2)
Antiplatelet	6 (2.8)
ACE inhibitor or ARB	1 (0.5)
Beta blocker	0 (0.0)
Calcium channel blocker	2 (0.9)
Statin	0 (0.0)
Total number of patients who underwent invasive procedures	39 (18.1)
Coronary angiography	39 (18.1)
Percutaneous coronary intervention	27 (12.6)
Coronary artery bypass graft surgery	10 (4.7)

Abbreviations: ACE, angiotensin-converting enzyme; ARBs, angiotensin receptor blocker; CMR, cardiac magnetic resonance.

## Discussion

In this retrospective cohort of patients with CAC undergoing stress perfusion CMR, several key findings were observed. First, the prevalence of CMR-detected myocardial ischemia was 26.0%. Second, CAC was the only factor independently associated with myocardial ischemia, with no heterogeneity across clinical risk score categories and diabetes status. Third, a CAC score ≥1,093 AU was potentially an optimal threshold associated with myocardial ischemia. Finally, The clinical impact of stress perfusion CMR included medication changes in 37.2% of patients, while 18.1% underwent invasive procedures.

The CAC score is a marker of coronary atherosclerosis and a well-established cardiovascular risk predictor that effectively reclassifies patients across risk categories [26]. Several guidelines endorse using the CAC score to improve risk assessment and guide LDL-C and non-HDL-C goals [27,28]. Clinical management based on CAC scoring was outlined in the ACC/AHA cholesterol guidelines, particularly regarding the initiation of lipid-lowering therapy and target LDL-C goals [28].

It is well established that patients with high CAC scores have a markedly increased risk of major adverse cardiovascular events [1–4]. Although a CAC score of zero offers an exceptionally high negative predictive value for ruling out myocardial ischemia and obstructive coronary artery disease (CAD), recent follow-up studies in initially zero-score populations show that subclinical CAC progression occurs in 23% of patients over 4.5 years [29], and 4% to 5% develop non-calcified obstructive disease or vulnerable plaques over 4 years [30]. Because underlying metabolic factors such as hypertension, elevated HbA1c, and body fat percentage can drive disease progression and subsequent ischemic risk despite a baseline zero score, clinical risk stratification should be personalized.

Beyond the initiation or titration of lipid-lowering therapy, downstream diagnostic investigations, such as stress testing following CAC scoring, have become a topic of interest. Recommendations regarding stress testing following CAC scoring in asymptomatic individuals remain heterogeneous across guidelines [31–33]. Several SPECT studies have investigated this clinical question. For instance, a meta-analysis by Bavishi et al. evaluated SPECT after CAC assessment in symptomatic and asymptomatic patients [13]. Among asymptomatic patients, the reported prevalence of inducible myocardial ischemia ranged from approximately 18% to 30% [13].

Our study provides additional evidence on the use of stress perfusion CMR in patients with CAC, an area with limited available data. The prevalence of myocardial ischemia in our study was relatively higher (26%). This can be explained by differences in patient risk profiles and potential variations between imaging modalities. The patients in our study were relatively older, with more than half being men. Approximately 75% had hypertension, 30% had diabetes mellitus, and the cohort exhibited an intermediate-to-high clinical risk profile. While SPECT is well-established for myocardial perfusion assessment, stress perfusion CMR provides high spatial resolution, facilitating the detection of subtle or subendocardial ischemic changes. Nevertheless, as our study was not designed to directly compare imaging modalities, the higher prevalence of ischemia observed in our cohort is best attributed to the high-risk profile of our study population rather than modality-specific performance.

In our study, the prevalence of myocardial ischemia was strongly related to the CAC score level; patients with a CAC score under 300 demonstrated no myocardial ischemia. Theses align with previous SPECT studies that established a low risk of abnormal SPECT findings below a CAC threshold of 400 AU [34]. Similar to studies by Miranda et al. [35] and Moser et al. [36], most perfusion defects below this threshold were minor and lacked prognostic significance. ROC analysis identified an exploratory CAC threshold of ≥1,093 AU with good sensitivity but modest specificity. Notably, adopting a pragmatic threshold of ≥1,000 AU preserves identical sensitivity with only a minor trade-off in specificity. While these hypothesis-generating cutoffs may assist in risk-stratifying candidates for stress testing and warrant prospective validation, fixed thresholds may not be directly applicable to specific subgroups, such as patients with diabetes mellitus, who typically present with more advanced subclinical atherosclerosis [37].

The PREVENT risk score and the Thai CV risk score demonstrated no difference in discrimination for stress CMR-derived myocardial ischemia when used as standalone tools. However, this finding may be limited by sample size and potential selection bias inherent in our study’s primary objective and enrollment criteria. Furthermore, both calculators are fundamentally designed to estimate long-term CV event probability from traditional systemic risk factors rather than directly detect functional myocardial ischemia. These findings highlight the complementary role of imaging, where CAC scoring provides independent risk refinement that remains significantly associated with stress CMR-derived ischemia across all clinical risk score categories.

Stress perfusion CMR also influenced clinical management in patients with CAC. Rather than a diagnosis of myocardial ischemia, approximately 10% demonstrated LGE that indicated a silent myocardial infarction (MI), since they did not report any known cardiovascular disease or typical symptoms. CMR-detected silent MI provides significant prognostic value for major adverse cardiac events and mortality [38] and carries a similar risk to recognized MI [39]. This warrants future studies of silent MI in this population with CAC. Regarding clinical impact, stress CMR prompted medication adjustments, predominantly antiplatelet agents and statins, in a substantial number of patients, optimizing CAD therapy and guiding invasive procedures in high-risk individuals. However, clinical management should be individualized rather than relying solely on CAC thresholds and CMR findings. Importantly, because all invasive procedures occurred in patients with CMR-detected ischemia, our study demonstrates an association between CMR findings and subsequent management decisions, rather than establishing direct causality or improved clinical outcomes, which warrants future study.

Our study has several limitations. First, this was a single-center study with a modest sample size, which may limit the generalizability of our findings. Second, our cohort consisted entirely of Southeast Asian patients, whose distinct risk profiles may not apply to other ethnic populations. Third, the retrospective, referral-based design introduces substantial selection bias, as only patients selectively referred for downstream stress perfusion CMR were included, rather than all individuals with CAC. Fourth, CMR interpreters were not blinded to CAC scores or baseline clinical characteristics, as image evaluations were derived from clinical reports. However, all interpretations were performed by CMR-trained cardiologists following standardized SCMR criteria for the diagnosis of myocardial ischemia. Finally, the ROC-derived CAC threshold of ≥1,093 AU was identified within a single-center cohort without external validation. Consequently, this cutoff should be regarded as exploratory and hypothesis-generating rather than a definitive, clinically actionable threshold until validated in larger, independent populations.

## Conclusions

In patients with CAC scores undergoing stress perfusion CMR, the prevalence of myocardial ischemia was approximately one-fourth. A high CAC score was independently associated with the presence of ischemia, and the degree of CAC burden directly correlated with ischemia severity. Stress CMR may influence clinical management in this population. Larger or prospective studies validating these findings and evaluating prognosis are warranted.

## Supporting information

S1 File Cardiac magnetic resonance (CMR) protocol. (DOCX)

S1 Table Clinical and imaging characteristics of the study population at the time of CMR, stratified by diabetes mellitus status. (DOCX)

## Abbreviations

AU: Agatston unit
BMI: body mass index
CAC: coronary artery calcium
CI: confidence interval
CMR: cardiac magnetic resonance
CT: computed tomography
LGE: late gadolinium enhancement
LV: left ventricular
LVEDV: left ventricular end-diastolic volume
LVEF: left ventricular ejection fraction
LVESV: left ventricular end-systolic volume
OR: odd ratio
PET: positron emission tomography
ROC: receiver operating characteristic
SPECT: single-photon emission computed tomography
