# Supplementary material for: Association between coronary artery calcium score and myocardial ischemia assessed by stress perfusion cardiac magnetic resonance imaging
Source: PLoS One. 2026 Oct 30;21(10):e0361659. doi: 10.1371/journal.pone.0361659 (PMC13722317; doi:10.1371/journal.pone.0361659)
Supplement: Supplementary file 1 [file pone.0361659.s001.docx]

**Cardiac magnetic resonance (CMR) protocol**

The CMR studies were performed with a 1.5 or 3.0 Tesla (T) Philips Achieva XR scanner (Philips Medical Systems, Best, The Netherlands). The images for the cardiac functional study were acquired using the steady-state free precession (SSFP) technique in multiple slice short-axis views, 2-chamber, 3-chamber, and 4-chamber views. Typical 1.5-T CMR parameters for cardiac functional assessment were as follows: echo time (TE) 1.8 milliseconds (ms), repetition time (TR) 3.7 ms, number of excitations 2, field of view (FOV) 390 × 312 mm, matrix size 256 × 240, reconstructed pixel size 1.52 × 1.21 mm, slice thickness 8 mm, and flip angle 70°.(1) The myocardial first-pass perfusion study involved a 4-minute infusion of adenosine at 140 μg/kg/min, followed by the injection of 0.05 mmol/kg of gadolinium contrast agent and a saline bolus, administered at a rate of 4 mL/s. Using an ECG-triggered, SSFP, inversion-recovery, single-shot, turbo gradient echo sequence, three short-axis slices at basal, mid, and apical left ventricular (LV) levels were obtained. Image parameters included a TE of 1.32 ms, TR of 2.6 ms, flip angle of 50 degrees, slice thickness of 8 mm, FOV of 270 mm, and reconstructed FOV of 320 mm.(1) Late gadolinium enhancement images were acquired 10 minutes after administering a second dose of gadolinium (0.1 mmol/kg, at a rate of 4 mL/s) using the 3D segmented-gradient-echo inversion-recovery sequence. These images were obtained in multiple short-axis slices, as well as in long-axis, 2-chamber, and 4-chamber views, similar to the functional images. Image parameters included a TE of 1.25 ms, TR of 4.1 ms, a flip angle of 15 degrees, FOV of 303 x 384 mm, matrix size of 240 x 256, in-plane resolution of 1.26 x 1.5 mm, slice thickness of 8 mm, and a sensitivity encoding factor of 1.5.(1)

**Reference**

1.	Pakdeesrisakda K, Kaolawanich Y. Prognostic value of stress perfusion cardiac magnetic resonance in patients with known or suspected coronary artery disease and supranormal left ventricular ejection fraction. Open Heart. 2026;13(1):e004086.
