# Supplementary material for: Association between coronary artery calcium score and myocardial ischemia assessed by stress perfusion cardiac magnetic resonance imaging
Source: PLoS One. 2026 Oct 30;21(10):e0361659. doi: 10.1371/journal.pone.0361659 (PMC13722317; doi:10.1371/journal.pone.0361659)
Supplement: Supplementary file 2 [file pone.0361659.s002.docx]

**S1 Table.** Clinical and imaging characteristics of the study population at the time of CMR, stratified by diabetes mellitus status.

|  | Diabetes mellitus | No diabetes mellitus | P-value |
| --- | --- | --- | --- |
|  | (n=72) | (n=143) |  |
| Age, years | 69.6 ± 8.1 | 69.2 ± 9.6 | 0.75 |
| Male | 42 (58.3) | 89 (62.2) | 0.58 |
| BMI, kg/m^2^ | 26.2 ± 4.7 | 24.1 ± 3.9 | ***0.001*** |
| Systolic BP, mmHg | 139.9 ± 18.8 | 136.8 ± 19.5 | 0.27 |
| Diastolic BP, mmHg | 72.9 ± 10.9 | 74.0 ± 11.2 | 0.47 |
| Heart rate, bpm | 86.0 ± 14.7 | 77.2 ± 13.8 | ***<0.001*** |
| CAD risk factors |  |  |  |
| Hypertension | 67 (93.1) | 94 (65.7) | ***<0.001*** |
| Hyperlipidemia | 62 (86.1) | 112 (78.3) | 0.17 |
| Family history of CAD | 1 (1.4) | 4 (2.8) | 0.66 |
| Current smoker | 3 (4.2) | 5 (3.5) | >0.99 |
| Former smoker | 3 (4.2) | 2 (1.4) | 0.34 |
| Medications |  |  |  |
| Antiplatelet | 34 (47.2) | 60 (42.0) | 0.46 |
| ACE inhibitor or ARB | 30 (41.7) | 42 (29.4) | 0.07 |
| Beta blocker | 37 (51.4) | 36 (25.2) | ***<0.001*** |
| Calcium channel blocker | 22 (30.6) | 33 (23.1) | 0.23 |
| Statin | 51 (70.8) | 98 (68.5) | 0.73 |
| Laboratory results |  |  |  |
| Total cholesterol, mg/dL | 148.1 ± 33.8 | 176.1 ± 40.8 | ***<0.001*** |
| Triglycerides, mg/dL | 136.9 ± 88.9 | 111.9 ± 62.0 | ***0.03*** |
| HDL cholesterol, mg/dL | 51.1 ± 12.9 | 58.7 ± 18.2 | ***0.003*** |
| LDL cholesterol, mg/dL | 71.1 ± 29.7 | 96.4 ± 38.8 | ***<0.001*** |
| eGFR, mL/min/1.73 m² | 77.1 ± 19.5 | 78.9 ± 18.3 | 0.51 |
| PREVENT risk score, mean ± SD, % ^a^ | 22.4 ± 7.6 | 16.5 ± 8.6 | ***<0.001*** |
| PREVENT risk score category, n (%) ^a^ |  |  | ***<0.001*** |
| Low/borderline risk | 0 (0.0) | 8 (6.8) |  |
| Intermediate risk | 1 (1.6) | 26 (22.2) |  |
| High risk | 62 (98.4) | 83 (70.9) |  |
| Thai CV risk score, mean ± SD, % ^b^ | 27.6 ± 4.8 | 21.6 ± 7.9 | ***<0.001*** |
| Thai CV risk score category, n (%) ^b^ |  |  | ***<0.001*** |
| Low risk | 0 (0.0) | 12 (9.8) |  |
| Intermediate risk | 5 (7.8) | 37 (30.3) |  |
| High/very high risk | 59 (92.2) | 73 (59.8) |  |
| CT |  |  |  |
| Total CAC score, Agatston units | 1,375.5 (649.5–2,144.4) | 800 (452–1,337) | ***<0.001*** |
| CAC score categories, n (%) |  |  | ***0.003*** |
| 1–99 | 0 (0.0) | 12 (8.4) |  |
| 100-299 | 3 (4.2) | 17 (11.9) |  |
| 300–999 | 25 (34.7) | 57 (39.9) |  |
| ≥1,000 | 44 (61.1) | 57 (39.9) |  |
| CMR |  |  |  |
| LVEDV index, mL/m² | 65.0 ± 13.3 | 68.9 ± 12.1 | ***0.03*** |
| LVESV index, mL/m² | 19.0 ± 9.2 | 19.8 ± 6.8 | 0.52 |
| LV mass index, g/m^2^ | 40.3 ± 10.3 | 39.9 ± 9.7 | 0.79 |
| LVEF, % | 71.5 ± 9.1 | 71.7 ± 7.1 | 0.89 |
| Presence of myocardial ischemia | 17 (23.6) | 39 (27.3) | 0.56 |
| Number of ischemic segments ^c^ | 5 (4–8) | 5 (3–9) | 0.95 |
| Presence of LGE | 7 (9.7) | 14 (9.8) | 0.98 |
| Number of LGE segments ^d^ | 3 (2–5) | 2.5 (1–4) | 0.48 |

A p-value<0.05 indicates statistical significance.

^a^ PREVENT risk score available for n=180.

^b^ Thai CV risk score available for n=186.

^c^ In patients with myocardial ischemia.

^d^ In patients with LGE.

**Abbreviations:** ACE, angiotensin-converting enzyme; ARB, angiotensin receptor blocker; BMI, body mass index; BP, blood pressure; CAC, coronary artery calcium; CAD, coronary artery disease; CMR, cardiac magnetic resonance; CT, computed tomography; CV, cardiovascular; eGFR, estimated glomerular filtration rate; HDL, high-density lipoprotein; LDL, low-density lipoprotein; LGE, late gadolinium enhancement; LV, left ventricular; LVEDV, left ventricular end-diastolic volume; LVEF, left ventricular ejection fraction; LVESV, left ventricular end-systolic volume; PREVENT, Predicting Risk of Cardiovascular Disease EVENTs.
